# Unrestricted kinematic alignment corrects fixed flexion contracture in robotically aligned total knees without raising the joint line in extension

**DOI:** 10.1186/s40634-023-00670-4

**Published:** 2023-11-11

**Authors:** Elliot Sappey-Marinier, Stefano Bini

**Affiliations:** 1 Department of Orthopaedic Surgery, Ramsay Santé, Hôpital Privé Jean Mermoz, Centre Orthopédique Santy, Lyon, 69008 France; 2 Department of Orthopaedic Surgery, University of California San Francisco, San Francisco, CA USA

**Keywords:** Personalized alignment, Fixed flexion deformity, Bone cuts, Robotically-assisted, Total knee arthroplasty, Caliper-based measure resection, Joint line level, Cruciate retaining

## Abstract

**Purpose:**

Mechanically Aligned Total Knee Arthroplasty (MA TKA) typically addresses fixed flexion contractures (FFC) by raising the joint line during extension. However, in unrestricted Kinematically Aligned TKA (KA TKA) utilizing a caliper-based resection technique, the joint line is not raised. This study aims to determine the efficacy of KA TKA in restoring full extension in patients with FFC without increasing distal femoral resection, considering tibial bone resection and sagittal component positioning.

**Methods:**

A retrospective study was conducted by a single surgeon, involving patients who underwent primary robotically assisted cruciate retaining unrestricted KA TKA between June 1, 2021, and December 1, 2022. Complete intraoperative resection and alignment data were recorded, including the thickness of distal femoral and proximal tibial bone cuts. Patients with a preoperative FFC ≥ 5° (study group) were compared to those with FFC < 5° (control group). The impact of variations in tibial resection and sagittal component positioning was assessed by comparing the heights of medial and lateral resections, sagittal femoral component flexion, and tibial slope. Group comparisons were analyzed using the Wilcoxon Signed Rank Test, with a significance level set at *p* < 0.05.

**Results:**

A total of 48 KA TKA procedures met the inclusion criteria, with 24 performed on women. The mean preoperative FFC in the study group was 11.2° (range: 5–25°), while the control group exhibited 1° (range: 0–4°) (*p* < 0.001). There were no statistically significant differences observed between the study and control groups in terms of distal femoral resections, both medially (*p* = 0.14) and laterally (*p* = 0.23), as well as tibial resection heights, both medially (*p* = 0.66) and laterally (*p* = 0.74). The alignment of the femoral component flexion and tibial slope was comparable between the two groups (*p* = 0.31 and *p* = 0.54, respectively). All patients achieved within 5 degrees of full extension at closure.

**Conclusion:**

Robotic arm-assisted unrestricted KA TKA effectively restores full extension without raising the joint line during extension for patients with a preoperative fixed flexion contracture.

**Level of evidence:**

III.

## Introduction

Fixed flexion contracture (FFC) refers to the inability to fully extend the knee in the sagittal plane. When total knee arthroplasty (TKA) is required, the incidence of FFC in knee osteoarthritis can be as high as 65% [51]. FFC has been associated with various negative outcomes, including increased anterior knee pain, poorer knee function, and increased energy requirements for activities such as walking and standing [9, 10, 38, 40, 53]. Thus, restoring full extension is an important goal of TKA.

In mechanically aligned (MA) TKA, the objective is to achieve a neutral alignment in both the coronal and sagittal planes [23]. A recent systematic review examined factors influencing the occurrence and management of FFC in TKA [3]. The review identified several intraoperative measures that address FFC, including soft tissue releases in the medial and posterior compartments, distal femoral resection, sagittal placement of the femoral component, and posterior condylar offset, which affects the posterior tibial slope [6, 8, 15, 17, 33, 34, 36, 45, 50, 54].

Over the past decade, personalized realignment strategies have gained attention to improve patient outcomes [18, 29, 41, 42]. One of these alternative methods is unrestricted, caliper-verified kinematic alignment (KA) [21, 22]. Unlike MA, KA aims to restore the pre-arthritic anatomy of the knee, disregarding the native alignment in relation to the mechanical axis, while respecting the native soft tissue envelope (thus eliminating the need for releases), soft tissue gaps, and joint line obliquity and height. In KA TKA, the knee is resurfaced by matching the thickness of the distal and posterior femoral resections to those of the condyles of the femoral component, accounting for missing cartilage and blade kerf [20]. KA technique avoids collateral ligament and posterior cruciate ligament (PCL) releases, potentially resulting in fewer complications, stiffness, instability, and revision surgeries [28, 44].

Regarding the correction of FFC, one study demonstrated that KA preserves bone and soft tissue compared to MA [4]. Furthermore, it has been shown that restoring FFC to within 5 degrees allows for full extension at one year without any noticeable clinical deficits [1, 39]. Additionally, numerous publications suggest that intraoperative correction to a perfectly straight knee is unnecessary [1, 50]. Based on our own experience and the observation that knees with 0–5 degrees of flexion intraoperatively and at one month regain native extension within a year, our senior author’s goal has been to restore extension within 5 degrees while maintaining a restored joint line and consistent medial gap at 0 and 90 degrees of flexion. However, to the best of our knowledge, no study has yet reported on the management of preoperative FFC using KA. Therefore, the objective of this study is to determine whether KA alignment, utilizing a robotically-assisted technique, restores intraoperative range of motion to within 5 degrees of full extension in patients with FFC, without raising the extension joint line. The hypothesis is that KA TKA will effectively correct FFC without raising the extension joint line, even in cases of severe preoperative FFC.

## Methods

A retrospective review was conducted of all consecutive primary robotically-assisted cruciate retaining (CR) KA TKAs performed by a single surgeon (S.A.B) using the MAKO platform and Triathlon Implant (Single radius CR femoral component with standard tibial baseplate and cruciate substituting insert, Stryker, Mahwah, New Jersey, USA) between June 2021 and December 2022. Complete intraoperative resection and alignment data were available for analysis. Patients included in the study met the medical necessity guidelines for TKA treatment set by the Centers for Medicare & Medicaid Services and had the following criteria: (1) Kellgren-Lawrence Grade III to IV osteoarthritis, (2) any severity of varus or valgus deformity, and (3) any severity of flexion contracture. Patients with prior knee fractures treated with open-reduction internal fixation, inflammatory or septic arthritis, or lower extremity neurologic disorders were excluded.

Preoperative patient characteristics such as age, sex, and body mass index (BMI) were collected from the clinical patient files. Intraoperative data was obtained using the navigation tools on the robot. Maximum flexion and extension angles, as well as alignment data (including hip-knee-ankle angle, mechanical lateral distal femoral angle (mLDFA), and medial proximal tibial angle (MPTA) [43]), and gap data were measured immediately after placing the femoral and tibial pins. All pins were placed intra-incisionally, and the patella was reduced prior to data collection. Maximum flexion was measured by holding the thigh in maximum flexion while allowing gravity to flex the tibia and bend the knee. No additional force was applied. Extension was measured with the hip and knee in a neutral position, and the leg was supported at the heel. No additional force was applied.

An unrestricted kinematic alignment approach was used, planned and performed using the software in a “bone mode” that corresponds to the subchondral bony surface. Therefore, these resections did not account for cartilage, and 2 mm [46] were added to the planned resections to match the implant thickness. For the specific prosthesis used, the femoral component had a thickness of 8.5 mm distally and posteriorly, and the tibial implant had a thickness of 9 mm with the thinnest polyethylene.

The size of the femoral implant was determined using a posterior referencing technique, selecting the size that best fit the patient’s anatomy while avoiding any mediolateral overhang and femoral notching. The component was flexed between 0 and nine degrees as necessary to achieve the best fit. Planned femoral bone resections, including the bone and kerf, were set at 6.5 mm distally on both medial and lateral compartments, regardless of coronal alignment. Planned femoral rotation resections were set parallel to the posterior condylar axis at 6.5 mm posteriorly on both medial and lateral compartments. The tibial implant was chosen to match the femoral component and cover the tibial plateau. Planned tibial resections were 7 mm on both medial and lateral compartments, matching the estimated native joint line obliquity, unless bone loss was observed. In cases of significant bone loss, a reference point was used to approximate the position where the bone loss exceeded the native joint line. The tibial baseplate rotation was determined based on a line bisecting the tibial eminence and parallel to a line bisecting the lateral tibial plateau, marked before making the tibial cut. However, with the knee trials in full extension, the tibial alignment was checked to ensure rotational alignment with the distal femoral component. This alignment was also compared to the prior mark and served as the final rotational landmark.

**Fig. 1:**
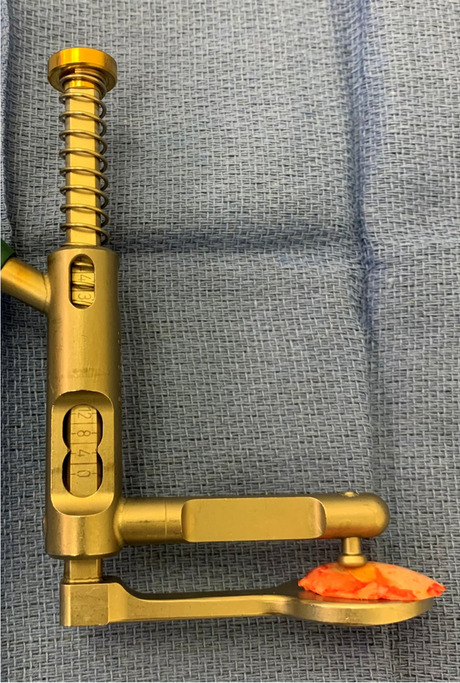
Caliper verification for femoral distal cut

A medial parapatellar approach was performed to expose the knee joint and remove osteophytes. The MAKO system of anatomical landmark registration was utilized, matching a 3D model of the implant to the patient’s preoperative CT scan and verifying bony anatomy accuracy within 1 mm [24, 47, 48]. Bone cuts were performed in the following sequence: posterior and anterior femoral cuts, tibial cut, distal femoral cut, and anterior and posterior chamfer femoral cuts. The distal femoral resection points were determined using the planning software and aligned sagitally with the peg holes of the femoral component. To ensure restoration of the joint line and account for any uneven wear of cartilage that could affect the perceived joint line or resection height, any remaining cartilage on the distal femoral condyles was removed to expose the subchondral bone using a sharp knife (subchondral referencing technique). Just before the resection, the marked distal resection point from the planning tool was identified using navigation. After performing the distal femoral cut and prior to the chamfer cuts, the resected bone wafer was measured using a flat caliper with an accuracy of 1 mm, using the mark on the cartilage and the back of the fragment as reference points. An average cartilage thickness of 2 mm and an additional 2 mm for the blade were taken into account to calculate the total resected bone (Fig. 1). The final resected height was then double-checked using a navigated planar probe, as described elsewhere [47].

Finally, trial components were inserted, and optical navigation from the MAKO system was used to measure the range of motion and laxity of the knee in maximum extension, employing the same techniques as described for collecting the pre-resection data. The sagittal plane balance was carefully assessed, aiming to achieve a range within 5 degrees of full extension. In the coronal plane, the goal was to obtain symmetric gaps in extension and a trapezoidal gap of 3 to 5 mm more in the lateral compartment at 90 degrees of flexion. Coronal plane balance was evaluated throughout the range of motion, and any imbalance in extension was addressed by adjusting the tibial cut into more varus or valgus, as needed, rather than resorting to soft tissue releases. If the extension and flexion gaps were not balanced, the additional bone resection was determined by the difference in the medial and lateral gaps, as measured in millimeters on the computer module with the knee at maximum extension and at 90 degrees of flexion. For instance, if the medial gap measured 17 mm and the lateral gap was 19 mm, the tibial cut was anchored to the lateral edge of the tibia, and the varus angle of the tibial cut was increased until the lateral and medial gaps were equal, in this case, by 2 mm. In the event of a tibial recut, the amount of bone recut determined based on the imbalance from the robot platform was added to the initial bone cut measurement. The thickness of the tibial cut corresponded either to the thickness of the initial tibial cut when no recut was performed or to the sum of the initial and recut tibia.

The study received approval from the scientific and ethics review committees (Institutional Review Board approval number 17–22672) and was conducted in accordance with the ethical standards of the institutional and/or national research committee and the 1964 Declaration of Helsinki and its subsequent amendments or comparable ethical standards.

Using a precise navigation tool, the fixed flexion contracture (FFC) threshold was set at 5 degrees. Patients with a preoperative FFC greater than or equal to 5 degrees (study group) were compared to those with FFC less than 5 degrees (control group). Intraoperatively, the thickness of the distal femoral and proximal tibial bone cuts, the sagittal positioning of the femoral and tibial components, the number of tibial recuts, and the maximum flexion and extension, along with the alignment data (including hip-knee-ankle angle, mechanical lateral distal femoral angle (mLDFA), and medial proximal tibial angle (MPTA)), were recorded. The dependent variables were reported as mean ± standard deviation (SD) using JMP Pro software, version 16.0.0. The potential impact of variations in tibial resection and component positioning in the sagittal plane was evaluated by comparing the medial and lateral resection heights, sagittal femoral component flexion, and tibial slope. The significance of the difference between patients categorized according to the preoperative FFC was determined using the Wilcoxon Signed Rank Test for all dependent variables. A sub-analysis compared femoral and tibial resections between patients with a preoperative FFC greater than or equal to 15 degrees and those with FFC less than 15 degrees. Statistical significance was set at *p* < 0.05.

## Results

A total of 48 cases of robotic arm-assisted kinematically aligned total knee arthroplasties with unrestricted caliper verification were analyzed. There were no significant differences in the proportion of female patients between the study group and the control group (61.9% vs. 40.7%, *p* = 0.14). The mean preoperative FFC in the study group was 11.2°, compared to 1° in the control group (*p* < 0.001). Table 1 summarizes all patients’ preoperative characteristics.

**Table 1:** Patient’s preoperative characteristics grouped by the preoperative fixed flexion contracture (FFC) (< 5° vs ≥ 5°)

Characteristics	Patients with FFC < 5° Means ± SD (range)	Patients with FFC ≥ 5° Means ± SD (range)	*P*-value
Number	21	27	
Age	67 ± 7 (53 to 84)	69 ± 6 (51 to 80)	0.41
Body-Mass-Index	29 ± 3 kg/m^2^ (26 to 38)	31 ± 4 kg/m^2^ (23 to 39)	0.53
Fixed Flexion Contracture	1° ± 1.6 (0 to 4°)	11.2° ± 5.7 (5 to 25°)	< 0.001
Maximum Knee Flexion	129° ± 8 (118 to 146°)	125° ± 10 (90 to 138°)	0.19
Coronal deformity (varus < 180, valgus > 180)	179° ± 3 (172 to 183)	177° ± 5 (171 to 188)	0.054

Between the study and control groups, there were no statistically significant differences in distal femoral resections, both medially (*p* = 0.14) and laterally (*p* = 0.23), or in tibial resection heights, both medially (*p* = 0.66) and laterally (*p* = 0.74) (Table 2). Table 3 presents the femoral and tibial resections comparing patients with a preoperative FFC greater than or equal to 15° to those with FFC less than 15°.

**Table 2:** Intraoperative distal femoral and tibial resection depths between both groups (fixed flexion contracture (FFC) < 5° vs ≥ 5°)

Resection depths (mm)	Patients with FFC < 5° Means ± SD (range)	Patients with FFC ≥ 5° Means ± SD (range)	*P*-value
Medial Distal Femoral	7.9 ± 0.7 (7 to 10)	8 ± 0.7 (5 to 9)	0.14
Lateral Distal Femoral	7.9 ± 0.7 (7 to 9)	8 ± 0.7 (6 to 10)	0.23
Medial Tibial	8.7 ± 1.1 (7 to 11)	8.5 ± 1.5 (6 to 11.5)	0.66
Lateral Tibial	8.9 ± 1.3 (7 to 11)	9 ± 1.4 (6 to 11.5)	0.74

**Table 3:** Sub analysis of intraoperative distal femoral and tibial resection depths between both groups (fixed flexion contracture (FFC) < 15° vs ≥ 15°)

Resection depths (mm)	Patients with FFC < 15° Means ± SD (range)	Patients with FFC ≥ 15° Means ± SD (range)	*P*-value
Number	42	6	
Medial Distal Femoral	7.9 ± 0.7 (5 to 10)	8.2 ± 0.4 (7.5 to 8.5)	0.26
Lateral Distal Femoral	8 ± 0.7 (6 to 10)	8 ± 0.4 (7.5 to 8.5)	0.67
Medial Tibial	8.5 ± 1.3 (6 to 11.5)	9 ± 1.2 (8 to 11)	0.35
Lateral Tibial	8.8 ± 1.3 (6 to 11)	9.5 ± 1.4 (8 to 11.5)	0.46

The alignment of the femoral component flexion and tibial slope was comparable between the study and control groups (*p* = 0.31 and *p* = 0.54, respectively). There was no correlation found between the sagittal position of either the femoral or tibial component and intraoperative FFC (*p* = 0.66 and *p* = 0.25, respectively). The number of tibial recuts was statistically similar between both groups (*p* = 0.38). At closure, all patients achieved a range of motion within 5 degrees of full extension. Intraoperatively, although a significant difference was observed for extension, it was not clinically significant (0.4° ± 1 vs. 1.5° ± 1.6, *p* < 0.001). Table 4 presents all patients’ intraoperative characteristics.

**Table 4:** Intraoperative coronal, sagittal components position and knee range of motion between both groups (fixed flexion contracture (FFC) < 5° vs ≥ 5°)

Intraoperative measurements	Patients with FFC < 5° Means ± SD (range)	Patients with FFC ≥ 5° Means ± SD (range)	*P*-value
Coronal deformity (varus < 180, valgus > 180)	180° ± 2 (177 to 184)	179° ± 3 (174 to 185)	0.16
Femoral component flexion	3.9° ± 2.1 (0 to 7.7°)	3.4° ± 2 (0 to 9.4°)	0.31
Tibial component slope	6.7° ± 2.2 (3.5 to 10.9°)	7.2° ± 2.1 (5 to 14.1°)	0.54
Sum of femoral and tibial sagittal positioning	10.7° ± 2.3 (7 to 15.2°)	10.6° ± 3.1 (6.9 to 23.5°)	0.79
Mechanical Lateral Distal Femoral angle (varus > 90, valgus < 90)	85.9° ± 2.6 (80.4 to 91)	87° ± 2.7 (81.5 to 92.5)	0.25
Medial Proximal Tibial angle (varus < 90, valgus > 90)	87.4° ± 2 (84.4 to 90.5)	87° ± 2 (82.4 to 89.3)	0.57
Number of tibial recuts	9	15	0.38
Maximum knee extension Contracture	0.4° ± 1 (0 to 4°)	1.5° ± 1.6 (0 to 5°)	< 0.001
Maximum Knee Flexion	131° ± 8 (120 to 149°)	130° ± 7 (117 to 141°)	0.68

## Discussion

The main finding of this study is that kinematic alignment for total knee arthroplasty can address fixed flexion contractures without needing to raise the extension joint line. Moreover, the femoral and tibial components’ sagittal positioning was similar between groups and did not impact the restoration of intraoperative knee extension. Secondary outcomes include restoration of coronal alignment and knee flexion.

To manage FFC when using MA techniques, several algorithms have been described. Those algorithms include soft tissue releases and if necessary, an additional 2 mm distal femoral bone resection to raise the extension joint line. A recent case control study [16] comparing 2634 MA TKAs showed that a systematic standardized algorithm for surgical treatment of flexion contracture during primary TKA provided clinical outcomes similar to those of patients without preoperative flexion contracture. The first step of the described algorithm, consisting in osteophytes removal and concavity release, allowed to correct most of FFC, assessed subjectively by the surgeon. However, the next step in case of persistent FFC, an additional 2 mm distal femoral bone resection was performed. Several studies, however, have shown that elevating the joint line can induce mid-flexion instability after TKA and should be avoided [6, 8, 11–14, 17, 25, 31, 33, 34, 45, 49, 52, 54]. Unrestricted KA aims to restore the patient’s anatomy prior to knee osteoarthritis without performing any soft tissue releases including the posterior cruciate ligament [19]. One study has shown that KA TKA is able to correct sagittal deformities with less bony resection and soft tissue releases to achieve the same amount of correction to full extension when compared to MA TKA [4]. To our knowledge, this is the first study comparing sagittal correction in patients with and without a preoperative FFC after KA TKA and no additional distal femoral bone resections were required to correct sagittal deformity when addressing FFC compared to patients without contractures.

Furthermore, soft tissue balancing remains one of the solutions described in the stepwise algorithms to manage FFC with MA. The present study found that no ligament releases were necessary to achieve a balanced and fully extended TKA according to KA principles. This has been widely shown after unrestricted KA TKA [2, 4, 26, 28]. As releasing the posterior cruciate ligament (PCL) increases both the flexion and the extension gap [37], many authors reporting on MA results have stated that patients presenting with a preoperative FFC could benefit from a posterior stabilized TKA [11, 14]. In contrast, the present study found that extension can be regained after PCL retaining KA TKA even in patients with a preoperative FFC.

Lombardi et al. [27] proposed a classification for fixed flexion contracture (FFC), where moderate FFC is defined as a contracture angle greater than 15°. In their stepwise algorithm for moderate to severe FFC, they recommended an additional 2 mm resection of the distal femoral condyle. Previous studies have shown that this additional 2 mm of distal femoral resection can resolve 10° of flexion contracture [7, 49], and when combined with soft tissue balancing, it can restore full extension in cases of moderate FFC after mechanical alignment (MA) total knee arthroplasty (TKA). However, in contrast to these findings, the present study did not find any difference in distal femoral resections for cases of moderate FFC when performing kinematic alignment (KA) TKA.

The optimal sagittal positioning of the femoral component has not been extensively studied. Two studies [30, 35] reported a higher proportion of post-operative FFC in knees with greater component flexion, as measured by computer navigation. However, the present study did not find any correlation between intraoperative knee extension and the sagittal position of the femoral component, which is consistent with another study [5].

There are several limitations to this study that may affect the generalizability of the results. Firstly, the findings are specific to the TKA design used in robotic-assisted, caliper-confirmed, unrestricted KA with a posterior cruciate ligament (PCL) retaining technique. Therefore, these results may not be applicable to other alignment methods or PCL sacrificing techniques, and further research is needed in those areas. Secondly, the number of patients with moderate intraoperative FFC (≥ 15°) was small, and the findings may not be applicable to this specific population or patients with even greater contractures. It is possible that residual contractures may remain in knees with contractures exceeding 15 degrees, likely in the range of 5 degrees. Previous research suggests that many residual contractures are due to soft tissue factors (such as hamstrings and posterior capsule) and tend to resolve over time as inflammation and pain subside [32]. Third, full extension was reached for the majority (58%) but not all patients. However, all knees extended within five degrees which is the goal of the senior author based on numerous publications showing that intraoperative correction to zero was not needed [1, 50] and that raising the extension joint line might be deleterious to long term outcomes. It is also important to note that navigation systems that use the femoral head as the reference point for sagittal alignment may induce error in the measurement of residual flexion contractures if there is femoral neck anteversion or retroversion. However, this introduces variation between but not within individual patients. Finally, this study did not report postoperative clinical outcomes or long-term range of motion as it was beyond the scope of the study, which aimed to determine whether unrestricted KA in TKA without raising the extension joint line could correct FFC.

## Conclusion

This study demonstrates that robotic arm-assisted kinematic alignment total knee arthroplasty can successfully restore full extension without the need to raise the joint line in patients with preoperative fixed flexion contracture, compared to those without FFC.
